# Disordered eating behavior, health and motives to exercise in young men: cross-sectional population-based MOPO study

**DOI:** 10.1186/s12889-016-3162-2

**Published:** 2016-06-08

**Authors:** Marjukka Nurkkala, Anna-Maria Keränen, Heli Koivumaa-Honkanen, Tiina M. Ikäheimo, Riikka Ahola, Riitta Pyky, Matti Mäntysaari, Raija Korpelainen

**Affiliations:** Department of Sports and Exercise Medicine, Oulu Deaconess Institute, P.O. BOX 365, FI-90101 Oulu, Finland; Center for Life Course Health Research, Faculty of Medicine, University of Oulu, P.O. BOX 5000, FI-90014 Oulu, Finland; Medical Research Center Oulu, University Hospital of Oulu and University of Oulu, P.O. BOX 5000, FI-90014 Oulu, Finland; Clinical Research Center, Internal Medicine, University Hospital of Oulu, University of Oulu, P.O. BOX 5000, FI-90014 Oulu, Finland; Department of Psychiatry, Institute of Clinical Medicine, Psychiatry, University of Eastern Finland, P.O. BOX 100, FI-70029 Kuopio, Finland; Clinic of Child Psychiatry, Oulu University Hospital, P.O. BOX 26, FI-90029 OYS Oulu, Finland; Departments of Psychiatry, Kuopio University Hospital (KUH), South Savonia Hospital District, Mikkeli; North Karelia Central Hospital, Joensuu; SOSTERI, Savonlinna; SOTE, Iisalmi; Lapland Hospital District, Rovaniemi, Finland; Center for Environmental and Respiratory Health Research, University of Oulu, P.O. BOX 5000, FI-90014 Oulu, Finland; Research Unit of Medical Imaging, Physics and Technology, Faculty of Medicine, University of Oulu, P.O. BOX 5000, FI-90014 Oulu, Finland

**Keywords:** Adolescent, Male, Disordered eating behavior, Physical activity, Motivation, Obesity

## Abstract

**Background:**

Being overweight is an increasing problem among young people, among whom disordered eating behavior is linked with weight problems as well as unhealthy weight control. The aim of the present study was to investigate whether health factors and motives to exercise differ in young men by the type of disordered eating behavior.

**Methods:**

The population-based, cross-sectional MOPO study consisted of 2,096 young Finnish men (mean age 17.9, SD 0.7) attending compulsory call-ups for military service in the Oulu area in 2010, 2011, and 2013. They responded to a questionnaire that included two subscales of the Eating Disorder Inventory-3 indicating drive for thinness and bulimic behavior and questions on health, physical activity, and motives to exercise. The association between disordered eating behavior and related factors was analyzed by binary logistic regression.

**Results:**

Altogether, 6.9 % (*n* = 145) of the men had symptoms of disordered eating, i.e., 5.4 % had a drive for thinness (*n* = 114) and 3.7 % had bulimic behavior (*n* = 77). Drive for thinness was associated with a perception of being overweight (OR 3.7; 95 % CI 2.2–6.1), poor self-rated health (2.3; 1.2–4.4), more leisure sitting time (1.1; 1.0–1.2), and body-related exercise motives (body acceptance: 3.0; 1.7–5.2; weight loss: 2.5; 1.4–4.4). Bulimic behavior was positively associated with poor self-rated health (2.6; 1.1–5.8) and several motives to exercise, i.e., due to another person’s suggestion (2.8; 1.6–4.8), competitive sports (2.1; 1.2–3.7), body acceptance (2.1; 1.1–3.9), and weight loss (1.9; 1.1–3.3), but inversely associated with health/fitness-related exercise motives (health promotion: 0.3; 0.1–0.5; muscular strength or physical performance: 0.5; 0.2–0.9).

**Conclusions:**

In young men, disordered eating behavior was associated with being overweight, having poor self-rated health, and having a greater amount of leisure sitting time as well as non-health-related motives to exercise. In order to recognize those at risk for disordered eating behavior, evaluating these factors could be beneficial.

## Background

Eating disorders are usually classified into the categories of anorexia nervosa, bulimia nervosa, and atypical eating disorders (eating disorders not otherwise specified), such as binge eating disorder. These disorders are much more common in women than in men. In Finland, less than 5 % of the patients treated in a specialized eating disorder clinic in 1995–2010 were men [1]. When abnormal eating behavior does not fulfill the criteria of an eating disorder, the term *disordered eating behavior* (DEB) is used.

DEB is associated with many co-problems, such as obesity [2], but also with several mental health problems, such as depressive symptoms [3–5], social phobia [6], and panic and anxiety disorders [5–7]. Furthermore, DEB tends to track from adolescence to adulthood and to later turn into an eating disorder [8, 9]. Thus, DEB, together with its comorbidities, can cause considerable adverse effects on an individual’s health and wellbeing as well as on society’s health care costs.

DEB is common among young people and adults; however, it is not as well-detected in men as it is in women [10, 11]. In previous studies, the prevalence of DEB among young men has varied, depending on the assessment method, from 6.5 % to 14 % [4, 5, 12], which is less than among young women (more than one quarter [5, 12]). However, many DEB questionnaires are based on female symptomatology, which may limit their ability to recognize male symptoms and result in reported lower prevalence among men. In men, DEB can appear as binge eating, restrictive eating due to negative emotions, or extreme and unhealthy weight control behaviors, e.g., skipping meals and use of food substitutes [12, 13]. It has also been positively associated with drive for muscularity, excessive exercise, and exercising to lose or gain weight and inversely associated with exercising for fun [10, 14]. Among male adolescents, compulsive exercise can result not only from a desire to be more muscular, especially from the waist up [15], but also from bulimic behavior or excessive exercise for losing weight for better health [10, 14]. However, DEB has not been related to the amount of physical activity in previous studies [14, 16, 17].

DEB has been associated with both measured and perceived overweight status [5, 12]. Compensatory dieting and extreme weight loss behaviors—which have been reported to increase in men from adolescence to young adulthood [18]—can be associated with future body mass increase in young men [19]. Moreover, dieting among young men may be more common if their body images are concurrently distorted [18] or if weight concerns were present earlier in adolescence [20].

In the young US male population, the prevalence of body image distortions—with or without binge eating or compensatory behavior—was as high as 33 % [9]. Their related traits are muscle and body dissatisfaction, which can also lead to a greater risk for DEB and depressive symptoms [21]. Up to 30 % of young Finnish men have been shown to be dissatisfied with their muscularity [22], which is more common in men than in women [23]. Body dissatisfaction has been found to be more prevalent among male adolescents who are under- or overweight than among those with normal weights [24].

Population-based studies on the role of physical activity and fitness in DEB and its different traits of drive for thinness and bulimic behavior among young men are lacking. However, such studies are needed for early identification of young men with disordered eating. The present study aims to investigate the prevalence of DEB and its relationships with health, physical activity, and motives to exercise. We hypothesized that DEB is positively associated with a high frequency of exercise, weight- and appearance-related exercise motives, and perception of being under- or overweight.

## Methods

### Study design and setting

The target population of the MOPO study consisted of all conscription-aged young men during 2009–2013 (*n* = 5,864) in the city of Oulu in Northern Finland, which has approximately 193,000 inhabitants. Data collection took place at the mandatory call-ups for military service arranged annually by the Finnish Defense Forces. Exclusion criteria to participate in the call-ups for military service included having a severe physical or mental illness that did not allow for independent living. The study included a medical examination before the call-ups and questionnaires and physiological measurements conducted during the call-up day. The aim of the study was to provide knowledge on measured physical activity and young men’s attitudes towards it, as well as health, fitness, nutrition, health information-seeking behavior, and other life habits and cultures among young men.

In this study, data collected during 2010, 2011, and 2013 that included information on disordered eating behavior. In total, 3,542 young men participated, of which 65.6 % (*n* = 2,322) took part in the physiological measurements and 70.3 % (*n* = 2,490) completed the questionnaire. Thus, the present study consisted of all those 2,096 (59.2 %) men (mean age of 17.9, SD 0.7 years) who responded to the questions on disordered eating behavior.

The procedures of the study were in accordance with the Declaration of Helsinki. The Ethics Committee of the Northern Ostrobothnia Hospital District approved the study design (ETTM123/2009), and written informed consent was obtained after a complete description of the study was given to the participants. Participation in the study did not affect the participants’ military service or their future health care.

### Study questionnaire

The study questionnaire included items on age, socio-economic status, disordered eating behavior, weight, health, physical activity, sedentary behavior, fitness, and motives to exercise.

### Disordered eating behavior

The participants were asked to respond to the two subscales of the Eating Disorder Inventory-3 questionnaire (EDI-3) as an indicator for DEB. One subscale described drive for thinness (DT), and the other described bulimic behavior (BB) [25]. Each subscale consisted of seven questions with a 6-point Likert scale from *never* to *always*, but the response alternatives *never* and *rarely* were combined for the analyses. Thus, the scale was scored as 0 = *never* or *rarely*; 1 = *sometimes*; 2 = *often*; 3 = *usually*; and 4 = *always* [25, 26]*.* The first question has an inverse scoring. The questionnaire has been validated for Danish women [26]. Cronbach’s alphas as a measure for internal consistencies were 0.74 for DT and 0.85 for BB.

### Weight

Perceived weight was determined from the responses to the following question: “What do you think about your weight?” A 5-point Likert scale was used for the response (1 = *very underweight* through 5 = *very overweight*). The response alternatives *very underweight* and *somewhat underweight* were combined, as was also done for *very overweight* and *somewhat overweight*. Weight history was investigated with the following questions: “Have you been overweight at some point of your life?” (1 = *yes, slightly overweight*; 2 = *yes, severely overweight*; 3 = *no*; and 4 = *do not know*, which was interpreted as never having been overweight for the analyses) and “Have you sometimes lost several kilos of weight?” (1 = *no*; 2 = *yes, weight loss was planned and controlled*; 3 = *yes, weight loss turned uncontrolled*).

### Health

Self-rated health was investigated with the following question: “How is the state of your health?” (1 = *good* through 5 = *poor*). The response alternatives *good* and *quite good* were combined, as was also done for *poor* and *quite poor*.

### Physical activity, sedentary behavior, and fitness

Daily physical activity was assessed with the following question: “How much do you approximately move during the day (e.g., while working, biking or walking to school or work, during the breaks at school, in household chores, or in hobbies and leisure time)?” (1 = *<1 h*; 2 = *1*–*2 h*; 3 = *>2 h*). Sedentary behavior was identified as daily sitting hours during leisure time with the following question: “How much do you sit per day outside school or work (e.g., watching TV, reading, using the computer, and driving a car)?” Self-rated physical fitness was assessed with the following question: “How fit do you think you are compared to your peers?” The response options used a 5-point scale (1 = *distinctly poorer* through 5 = *significantly better*). The response alternatives *distinctly poorer* and *somewhat poorer* were combined, as was also done for *significantly better* and *somewhat better*.

### Motives to exercise

The motives to exercise were investigated by a modified version of Nigg’s question [27]: “Are the following issues important for your exercising?” The response choices given were the binary options *yes* or *no*. The motives to exercise were classified according to Markland and Ingledew [28] and Ingledew and Markland [29] into four categories:

- *body* (body acceptance, enhancing appearance, increasing sex appeal, weight loss);
- *health/fitness* (enhancing muscle mass, improving or maintaining muscular strength and/or physical performance, promoting health, reducing stress);
- *social engagement* (competing and succeeding in athletics or sports, suggestion of a friend or family member, helping relationships, improving one’s respect from peers); and
- *enjoyment* (enjoying the feelings of euphoria gained by exercise, enjoying heavy exertion, increasing energy, improving spirits).

### Measurements

The physiological measurements included anthropometry (height, body weight, body mass index, body composition), maximal isometric hand grip strength (as a measure of upper body strength) and aerobic fitness assessments.

Height was measured with a wall-mounted measuring tape and was recorded at a 0.5-cm precision. Body composition (weight with 0.1 kg accuracy, body mass index (BMI), fat free mass in kilograms at 0.1 kg accuracy, percentage body fat) was measured using bioelectrical impedance analysis (InBody 720 device, Biospace Co., Ltd., Seoul, Korea) with the participant standing without shoes and socks and wearing lightweight indoor clothes. BMI was classified according to the World Health Organization guidelines: underweight (<18.5), normal (18.5–24.9), and overweight (≥25) [30]. The percentage of fat free mass was calculated by dividing fat free mass by weight.

Grip strength (kg) was measured using a dynamometer (Saehan, SAEHAN Corporation, Korea). During the examination, the participant stood with his legs apart and his elbow at a 90° angle and was instructed to grip the instrument with maximum strength. The measurement was repeated, and the higher result was selected. The mean of both hands was used in the analyses.

Aerobic fitness was assessed by the Polar Fitness Test™ (Polar Electro, Finland), which estimates maximal oxygen uptake (mL/min/kg) based on resting heart rate, heart rate variability, gender, age, height, body weight, and self-assessed physical activity [31].

### Statistical analysis

The cut-off scores for DEB were based on the 95^th^ percentile in the Drive for Thinness [32]. This cut-off has also been previously used for the EDI-2 subscales in a sample of adolescents [33]. Thus, a score of ≥11 was classified as having DT. In the Bulimia subscale, the 95^th^ percentile was at 7 points, but because 7 points could be earned by answering *sometimes* to each question, those having ≥8 points were classified as having BB.

The group differences between those with and without DEB (DT and/or BB) were compared with the independent samples *t*-test or with the nonparametric Mann–Whitney *U* test. The Chi-square test was used for categorical variables. The level of significance was retained at the p-value <0.05. All variables significantly associated with DEB in the univariate analysis were entered in the binary forward stepwise logistic regression. The results are presented as odds ratios (OR) with 95 % confidence intervals. The Nagelkerke R-square was used for explaining the variance of risk of having a DEB trait. Statistical analyses were performed using PASW Statistics 18 for Windows.

## Results

The characteristics of the total study population (*n* = 2,096) and those with and without DEB are described in Table 1. Eighty-six percent (*n* = 1,800) of the participants were students, 3 % (*n* = 61) were workers, 8 % (*n* = 159) were both, and 3 % (*n* = 61) were unemployed. The data on physiological measurements were available from a total of 1,614 participants.

**Table 1:** Characteristics of the young men with drive for thinness (DT) or bulimic behavior (BB) compared to the men without disordered eating behavior (DEB)

Characteristics	All (*n* = 2,096)	DT (*n* = 114)	BB (*n* = 77)	No DEB (*n* = 1,951)
Age, years	17.9 (0.7)	18.1 (1.0)	18.0 (0.8)	17.9 (0.6)
Height, cm	177.9 (6.4)	178.3 (5.4)	178.3 (5.1)	177.9 (6.4)
Weight, kg	72.5 (13.5)	82.9 (15.8)***	74.4 (16.9)	72.0 (13.1)
Body Mass Index, kg/m^2^	22.9 (4.0)	26.1 (4.9)***	23.4 (4.9)	22.7 (3.8)
Underweight, n (%)	129 (8)	1 (1)*	3 (6)	126 (8)
Overweight, n (%)	373 (23)	44 (57)***	12 (23)	326 (22)
Body fat percentage (%), median (95 % CI)	14.2 (13.8; 14.7)	22.4 (19.1; 26.3)***	13.7 (12.3; 15.8)	14.1 (13.7; 14.5)
Fat free mass (%), median (95 % CI)	48.3 (48.1; 48.5)	43.8 (41.8; 45.5)***	48.6 (47.8; 49.5)	48.4 (48.2; 48.6)
Aerobic fitness, mL/min/kg	53.6 (7.4)	50.5 (8.5)***	54.3 (8.3)	53.7 (7.2)
Grip strength, kg	46.6 (8.1)	47.9 (9.0)	48.6 (7.7)	46.5 (8.1)
Sitting hours (leisure time)	3.9 (2.2)	4.7 (2.5)**	4.3 (2.5)	3.9 (2.2)
Self-rated fitness poorer than among peers, n (%)	502 (24)	54 (48)***	24 (31)	439 (23)
Daily physical activity				
<1 hour, n (%)	487 (24)	30 (27)	16 (21)	449 (24)
1–2 hours, n (%)	1038 (51)	55 (50)	38 (49)	970 (51)
>2 hours, n (%)	522 (26)	25 (23)	23 (30)	487 (26)
Poor self-rated health, n (%)	107 (5)	18 (16)***	10 (14)**	87 (5)
Perceived weight				
Underweight, n (%)	349 (17)	11 (10)*	11 (15)	333 (17)
Ideal weight, n (%)	1288 (62)	32 (29)***	44 (59)	1235 (64)
Overweight, n (%)	425 (21)	67 (61)***	20 (27)	353 (18)
History of being overweight, n (%)	657 (32)	70 (65)***	33 (44)*	577 (30)
History of weight loss, n (%)	448 (22)	51 (47)***	25 (33)**	389 (20)

*Note:* One participant can have one or both DEB. Numbers do not match due to missing values

*SD* Standard Deviation, *CI* Confidence Intervals

*p<0.05; **p<0.01; ***p<0.001, reference group: no DEB

Values are means (SD) unless otherwise stated

Those who did not complete (*n* = 1,446) the study questionnaire did not differ from the study participants regarding perceived weight, self-rated health, sedentary behavior, and anthropometric measures. However, they were less frequently students (76 % vs. 86 %, *p* < 0.001), and they rated their fitness less frequently as good compared to their peers (29 % vs. 38 %) (*p* = 0.001).

Altogether, 145 (6.9 %) young men had either a drive for thinness (114/5.4 %) or bulimic behavior (77/3.7 %). Forty-six (2.2 %) had both DT and BB. Young men with DT more frequently had poor self-rated health and poorer physical fitness (both self-rated and measured), were on average 10.9 kg (95 % CI 7.2; 14.5) heavier, had higher body fat percentages (medians 22.4, 95 % CI 19.1; 26.3 vs. 14.1, 95 % CI 13.7; 14.5) and lower fat free mass (medians 43.8, 95 % CI 41.8; 45.5 vs. 48.4, 95 % CI 48.2; 48.6), and had a history of being overweight and/or losing weight more frequently than those without DEB (Table 1). The most significant differences in the motives to exercise by DT status were related to motives due to weight loss, body acceptance, and suggestions from friends or family members (Table 2). The young men with BB more often had poor self-rated health and a history of being overweight and/or losing weight than those without DEB (Table 1). They differed from young men without DEB in motives to exercise, for example, due to suggestion from others, respect from peers, weight loss, and body acceptance (Table 2).

**Table 2:** Motives to exercise in the young men with drive for thinness (DT) or with bulimic behavior (BB) compared to the men without disordered eating behavior (DEB)

	All (*n* = 2,096)	DT (*n* = 114)	BB (*n* = 77)	No DEB (*n* = 1,951)
	n (%)	n (%)	n (%)	n (%)
Health/fitness-related				
Promoting health	1753 (86)	97 (87)	56 (74)**	1635 (86)
Improving or maintaining muscular strength and/or physical performance	1642 (81)	82 (75)	52 (68)**	1539 (81)
Enhancing muscle mass	1470 (72)	74 (67)	56 (74)	1373 (72)
Reducing stress	1140 (56)	74 (67)*	46 (61)	1046 (55)
Enjoyment-related				
Enjoying the feelings of euphoria gained by exercise	1542 (76)	87 (80)	58 (77)	1430 (76)
Increasing energy	1473 (73)	89 (81)*	55 (74)	1361 (72)
Improving spirits	1469 (72)	83 (76)	54 (72)	1364 (72)
Enjoying heavy exertion	1093 (54)	69 (63)*	42 (57)	1007 (54)
Body-related				
Enhancing appearance	1281 (63)	86 (78)**	44 (58)	1178 (63)
Body acceptance	1147 (57)	91 (82)***	55 (73)**	1033 (55)
Increasing sex appeal	1021 (51)	72 (64)**	42 (55)	929 (50)
Weight loss	731 (36)	84 (76)***	39 (51)**	634 (34)
Social engagement-related				
Helping relationships	1090 (54)	62 (58)	44 (60)	1010 (54)
Improving respect from peers	754 (37)	55 (51)**	39 (52)**	681 (36)
Competing and succeeding in athletics or sports	694 (34)	33 (30)	36 (47)*	645 (34)
Suggestion of a friend or family member	391 (19)	39 (36)***	31 (42)***	339 (18)

*Note:* One participant can have one or both DEB. Numbers do not match due to missing values

*p<0.05; **p<0.01; ***p<0.001, reference group: no DEB

### Factors associated with drive for thinness

According to the multivariable logistic regression analysis, self-perception of being overweight, poor self-rated health, leisure time sitting hours, and having body acceptance and weight loss as motives to exercise were positively and significantly associated with the risk of having DT in young men (Table 3). The model including all these factors explained 21 % of the risk of having DT.

**Table 3:** Factors associated with drive for thinness (DT) in young men (*n* = 1,801) according to the multivariate logistic regression analysis

Variable	Adjusted OR (95 % CI)	*P*
Model R^2^ = 0.208^a^		
Leisure time sitting hours, per 1 hour increment	1.10 (1.00–1.20)	0.046
Perception of being overweight (vs. underweight or ideal weight)	3.65 (2.20–6.06)	<0.001
Poor self-rated health (vs. good or average)	2.32 (1.22–4.41)	0.010
Motives to exercise		
weight loss	2.45 (1.37–4.36)	0.002
body acceptance	2.98 (1.70–5.23)	<0.001

*Note:* Variables significant (*p* <0.05) in univariate analysis and DT were adjusted for in the forward stepwise logistic regression analysis

*OR* Odds Ratio, *CI* Confidence Interval

^a^R^2^ = Nagelkerke

Reference group: those without DEB

### Factors associated with bulimic behavior

According to the multivariate logistic regression analysis, poor self-rated health and motives to exercise due to suggestions from others, competition, weight loss, and body acceptance were positively and significantly associated with the risk of having BB in young men (Table 4), whereas having health promotion or improvement/maintenance of muscular strength/physical performance as the motives to exercise were negatively and significantly associated with the risk of having BB. The model including all these factors explained 13 % of the risk of having BB.

**Table 4:** Factors associated with bulimic behavior (BB) in young men (*n* = 1,865) according to the multivariate logistic regression analysis

Variable	Adjusted OR (95 % CI)	*P*
Model R^2^ = 0.131^a^		
Poor self-rated health (vs. good or average)	2.56 (1.14–5.75)	0.023
Motives to exercise		
competing and succeeding in athletics or sports	2.13 (1.22–3.72)	0.008
improving or maintaining muscular strength and/or physical performance	0.45 (0.23–0.87)	0.017
weight loss	1.91 (1.09–3.34)	0.023
promoting health	0.25 (0.13–0.50)	<0.001
body acceptance	2.06 (1.08–3.91)	0.027
suggestion of a friend or family member	2.78 (1.61–4.82)	<0.001

*Note:* Variables significant (*p* <0.05) in univariate analysis and BB were adjusted for in the forward stepwise logistic regression analysis

*OR* Odds Ratio, *CI* Confidence Interval

^a^R2 = Nagelkerke

Reference group: those without DEB

## Discussion

Our study showed that among young men, body-related motives to exercise were positively associated with disordered eating behavior (DEB), regardless of DEB’s trait. In addition, bulimic behavior (BB) was positively associated with motives related to competition in sports or suggestions from others but inversely associated with health/fitness-related motives. Perception of being overweight, poor self-rated health, and more leisure sitting time were positively associated with drive for thinness (DT), but only poor self-rated health was linked with BB.

In general, the prevalence of DEB was 6.9 % in the current study, which is consistent with previously reported prevalences of disordered eating and extreme weight control behaviors (from 6.5 % to 14 %) [4, 5, 12]. In this representative sample of young men, DT was the main type of disordered eating behavior. The prevalences in the present study (5.4 % for DT and 3.7 % for BB) are in accordance with a previous study [4]. However, a contrasting prevalence of DT and BB among young men has also been recently reported: 3.4 % for DT and 5.8 % for BB [34].

In the present study, DT was most prevalent among men who were subjectively or objectively perceived as being overweight. This is in accordance with a Finnish population-based twin study of men, in which larger body size was associated with higher DT scores, which, on the other hand, were associated with both overeating and restrictive eating [35]. Thus, DT may refer to aiming at weight loss, not objective thinness, as has been previously thought. This is also consistent with a previous study among young adult women [36]. In our obesogenic culture, it is common and even preferred to aim at becoming thinner, particularly if one is overweight. Also, men have been reported to strive for leanness and muscularity rather than thinness itself [10]. Even if DT may be a somewhat misleading term in this connection, most importantly, it can predict an eating disorder among young men [37]. More complicated is the thin line between normal and abnormal behavior and at what point DT turns into compulsive behavior that constricts one’s normal life.

According to our results, DT was also associated with poor self-rated health and more leisure sitting time. In a previous study, DT was associated with muscle dissatisfaction, which, in turn, was linked with a sedentary lifestyle [22]. In our study, DT was also associated with motives to exercise related to weight loss and body acceptance. Consistently, previous studies have demonstrated that exercising because of weight loss and attractiveness was associated with DT [14, 38]. Bratland-Sanda et al. suggested that these motives to exercise mainly stem from extrinsic factors instead of exercise itself. It is also possible that the social environment and media may cause pressure to lose weight and enhance appearance [39].

In the present study, poor self-rated health and several different motives to exercise (i.e., competition/success in sports, weight loss, body acceptance, and suggestions from others) were associated with having BB. On the contrary, the motives to exercise due to health benefits (i.e., health promotion, and muscular strength or physical performance) were inversely associated with having BB. In a recent study, BB and DEB were also associated with body-related (weight and appearance management) exercise motives [17], whereas those without DEB have been found to exercise more often for fun [14], indicating subjective wellbeing and constructive, intrinsic motives to exercise.

In our study, BB was unrelated to current weight—either under- or overweight. The finding is surprising compared to previously reported results that men are motivated to lose or gain weight because of, for example, athletic achievement [10], which is a risk factor for eating disorders in men [13]. Although current weight was not associated with BB in young men, weight loss and/or body acceptance as motives to exercise and a history of being overweight or losing weight were related to BB. Having previously been overweight, as was the case for one-third of our study participants with BB, has been shown to be associated with the development of eating disorders [10]. Also, due to recent growth spurts, binge eating may be hidden among young men who don’t have any overweight issues as of yet.

Furthermore, the amount of physical activity was not associated with DEB subscales, either in the present or in previous studies [14, 17], but low weekly physical activity was linked with body dissatisfaction [14]. Leisure sitting time was positively associated with DT in this study, which might also indicate a lower level of leisure physical activity.

The strength of our study is its population-based design with a representative sample of young men. Even if the DEB data was lacking from 40 % of the study population, comparison between responders and non-responders of the DEB questionnaires did not reveal differences in the essential study variables (weight, perceived weight, sedentary behavior, self-rated health). Furthermore, there are no previous studies investigating DEB traits among young men based on the EDI-3. A Finnish version of the EDI-2 with these subscales, but with somewhat different (more concise) scoring, has also been used [22, 35]; however, it has been criticized for being poorly suited for non-clinical populations [26]. Still, a limitation is that the EDI-3 questionnaire has been developed to assess eating disorders [in girls, not in young men], not DEB or its early and slight risks. Thus, there is lack of validation of the EDI-3 as a disordered eating behavior questionnaire in men, which is why validated cut-off scores do not exist. Also, the questionnaire lacks items that could be relevant to DEB among young men, such as muscle and body dissatisfaction [15, 21], sexuality [40], engaging in exercise as a compensatory behavior, and the desire to be lean instead of being thin [10].

Research is needed to help parents and various professionals in educational or health-care sectors to recognize DEB. Paying attention to symptoms of DEB in schools or during health check-ups, especially among overweight and obese young men with poor self-rated health, could be important for early prevention of adverse effects on health and wellbeing. Also, in physical education and organized sports, motives to exercise should be monitored, especially if they are purely body-related. The result of the present study may also benefit school-based interventions reducing DEB and preventing excess weight.

## Conclusions

In this population-based study among young men, DEB was associated with non-health-related motives to exercise, such as competitive sports, weight loss, and body acceptance. Thus, early identification of young men with DEB may benefit from evaluating motives to exercise in addition to evaluation of sedentary behavior and self-perceptions of perceived weight and health. Development and validation of the Eating Disorder Inventory-3 subscales among young men is needed.

## Abbreviations

BB, Bulimic behavior; BMI, Body mass index; CI, Confidence interval; DEB, Disordered eating behavior; DT, Drive for thinness; EDI-3, Eating Disorder Inventory-3; OR, Odds ratio; SD, Standard deviation
